# On the Possible Nature of Armchair-Zigzag Structure Formation and Heat Capacity Decrease in MWCNTs

**DOI:** 10.3390/ma15020518

**Published:** 2022-01-10

**Authors:** Alexander Ponomarev, Valeriy Egorushkin, Nadezhda Bobenko, Maksym Barabashko, Anastasiya Rezvanova, Anna Belosludtseva

**Affiliations:** 1 Institute of Strength Physics and Materials Science of the Siberian Branch of the Russian Academy of Sciences (ISPMS SB RAS), 2/4, pr. Akademicheskii, 634055 Tomsk, Russia; alex@ispms.ru (A.P.); root@ispms.tomsk.ru (V.E.); ranast@ispms.tsc.ru (A.R.); anna.bel@ispms.ru (A.B.); 2 B. Verkin Institute for Low Temperature Physics and Engineering of the National Academy of Sciences of Ukraine (B. Verkin ILTPE NASU), 47 Nauky Ave., 61103 Kharkov, Ukraine; msbarabashko@gmail.com

**Keywords:** structural disorder, carbon nanotubes, domain structure, heat capacity, phonons, size effect

## Abstract

Structural disorder and temperature behavior of specific heat in multi walled carbon nanotubes (MWCNTs) have been investigated. The results of X-ray diffractometry, Raman spectroscopy, and transmission electron microscopy (TEM) images are analyzed. The thermodynamic theory of the zigzag-armchair domain structure formation during nanotube synthesis is developed. The influence of structural disorder on the temperature behavior of specific heat is investigated. The size of domains was estimated at ~40 nm. A decrease in heat capacity is due to this size effect. The revealed dependence of the heat capacity of MWCNTs on the structural disorder allows control over thermal properties of nanotubes and can be useful for the development of thermoelectric, thermal interface materials and nanofluids based on them.

## 1. Introduction

Carbon nanotubes (CNTs) are the new generation of materials with a wide range of technological applications, one of which is the production of thermoelectric [1] and thermal interface materials [2], nanofluids [3], etc. designed for energy efficiency. In this case, the CNT structure plays an important role. Structural features depend on the synthesis methods, among which are arc discharge (AD), laser vaporization (LV), and chemical vapor deposition (CVD) [4]. The CVD method is most widely used due to its simplicity, ability to produce materials with more than 95% CNTs, low energy consumption, and the possibility of controlling the structure via the synthesis mode [5].

The physical and chemical properties of nanotubes are determined by their structure. The CNT surface is composed of graphene fragments (crystallites) [6]. Their type (armchair, zigzag), size, and orientation are the most important characteristics of structural disorder of nanotubes [6,7,8].

The armchair-zigzag boundaries were discussed in terms of energy efficiency and defect kinetics in graphene [9,10,11]. It was found that asymmetric boundaries are more energetically favorable than symmetric ones due to effectively reduced mechanical stresses.

Multi-walled carbon nanotubes (MWCNTs) prepared by the CVD method are usually metallic, while single-walled tubes (SWCNTs) are semiconductive or metallic. Both semiconductive and metallic nanotubes with high heat capacity and thermal conductivity are required for practical applications [12]. The control over these properties is determined by the possibility of optimizing heat capacity and thermal conductivity depending on the CNT structure. The structure–heat capacity relationship and its physical understanding are also important issues [13].

The experimental study of the specific heat for CNTs was carried out in refs. [13,14,15,16,17,18,19,20]. The authors of ref. [17] found that the heat capacities of MWCNTs and bundles of single wall carbon nanotubes (SWCNTs) are close to the values for graphite and diamond at near-room temperatures. The influence of the structure (its type (mosaic or bamboo one), the number and length of walls) on the heat capacity of CNTs becomes pronounced at temperatures below 150 K [15]. It was found that, with increasing temperature, the character of the temperature dependence for both MWCNTs [13,16,17] and SWCNTs [14] changes from $\sim T^{3}$ to $\sim T^{1}$.

An explanation of the temperature dependence *C*(*T*) for layered structures with weak coupling between them without any disorder was predicted in [21,22]. The explanation was based on the change of the dispersion law of phonon modes with temperature. The influence of structural disorder (armchair-zigzag one) on the heat capacity of graphene nanoribbons was studied in [23]. It was found that the width and disorder of boundaries influence significantly the *C*(*T*) dependence. In this case, the phonon dispersion law is similar for both types of boundaries. Other theoretical models describing the *C*(*T*) dependence are consistent with the experiment in certain temperature ranges [24,25,26].

In this work, we experimentally study the structure (X-ray diffraction and Raman spectroscopy), discuss TEM images and temperature dependences of the specific heat of SWCNTs and MWCNTs. Consideration will be given to a possible mechanism of the disordered structure formation in armchair-zigzag CNTs and the effect of structural disorder on the heat capacity in MWCNTs in comparison to *C*(*T*) for SWCNTs.

## 2. Materials and Methods

MWCNTs were prepared using the CVD method by decomposing ethylene over the bimetallic Fe-Co/Al_2_O_3_ catalyst at 670 °C [27]. After deposition, nanotubes were treated with 15% HCl and then washed with distilled water until neutral pH [27]. The average diameter of nanotubes is 18 nm, the average length of MWCNTs is 30 μm, and the density is ~2 g/cm^3^. The amount of carbon is ~99 wt%, and catalyst particles are near 0.3–0.5 wt% [17]. This catalyst composition provides MWCNTs with low defectiveness and with a low content of inorganic impurities, as was clarified by the X-Ray photoelectron spectroscopy (XPS) and Near-edge X-ray absorption fine structure (NEXAFS) measurements made at the Russian German Photoemission Station of Russian Germany Dipole Beamline of the 3rd generation synchrotron radiation source BESSY II (Berlin, Germany) [16]. The specific heat of MWCNTs was measured [17] in the temperature range from 1.8 to 150 K by the thermal relaxation method using the physical property measurement system (PPMS) by Quantum Design Inc (San Diego, CA, USA).

The structure and chemical composition of MWCNTs were studied by TEM, Raman spectroscopy, and X-ray diffraction techniques. X-ray diffractometry was performed with a high-resolution Bruker Discover D8 diffractometer (Billerica, MA, USA) with Cu*K*α radiation, λ = 1.5406 Å. The phase composition was estimated using the DIFFRAC.EVA (version 1.3.1, Bruker Optics Inc, Billerica, MA, USA), and the full profile analysis was made using the POWDER CELL software (version 2.3, Federal Institute for Materials Research and Testing, Berlin, Germany). TEM was carried out through a JEOL JEM-2010 electron microscope (JEOL Ltd., Tokyo, Japan) with the lattice accelerating potential 200 kV [17]. Raman spectroscopy was carried out at the Scientific Park of NR TPU using the NT-MDT-Solar AFM/Raman spectrometer (NT-MDT, Moscow, Russia) at the wavelength of the exciting laser radiation 633 nm and 100× magnification.

The theory of phase transitions, crystal symmetry, and the Green function method was employed to study the effect of structural changes in CNTs on their thermal properties [28,29,30].

## 3. Results and Discussion of the Armchair-Zigzag Structure of MWCNTs

**Figure 1:**
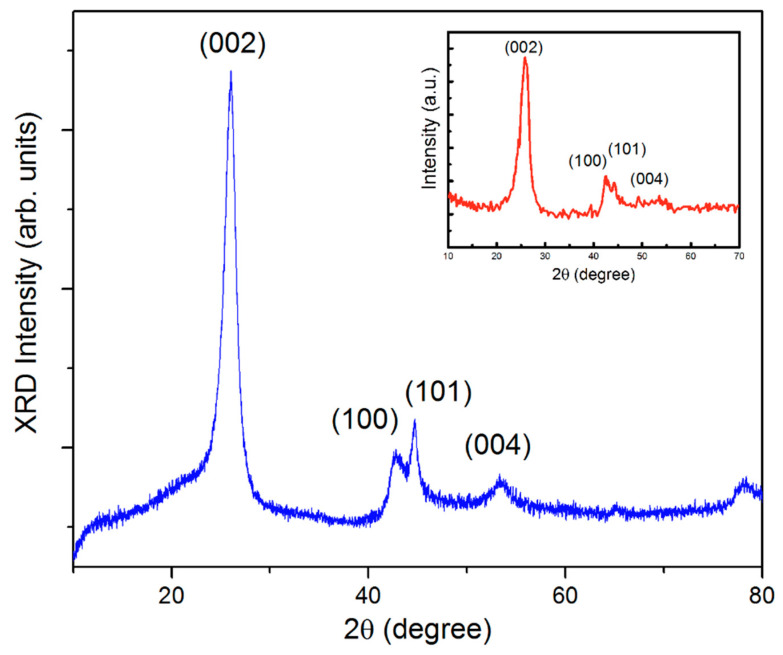
X-ray pattern for MWCNTs. The insert shows the X-ray pattern for SWCNT bundles [31].

The X-ray diffraction(XRD) data for SWCNTs [31] and MWCNTs are presented in Figure 1. Both types of nanotubes show (002) and (100), (101), and (004) peaks, which are assigned to the hexagonal ring structure of graphite sheets forming carbon nanotubes [32,33].

The lower insert schematizes the domain structure of MWCNTs.

**Figure 2:**
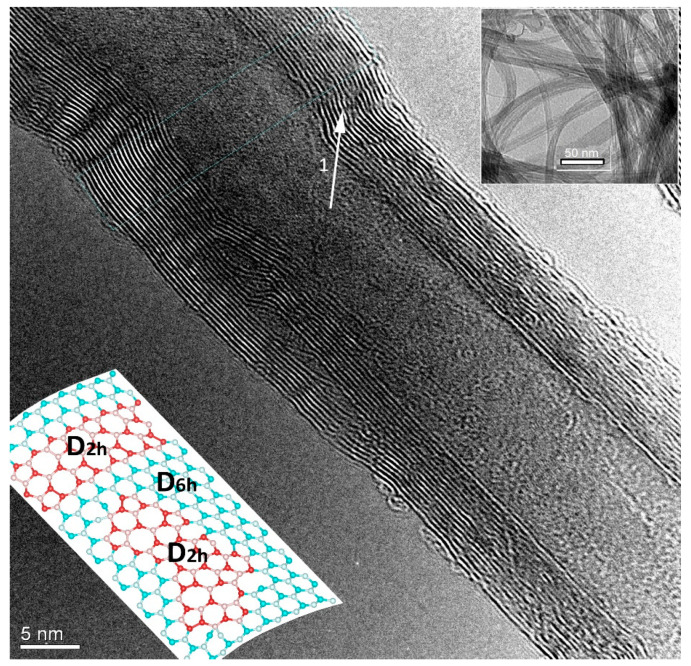
High-resolution TEM images of MWCNTs and SWCNTs (the upper insert).

Figure 2 illustrates the morphology of MWCNTs. The average diameter (d) of tubes is found to be 18 nm, and the average number of walls is ~15. Arrow 1 points to the boundary between differently oriented hexagonal crystallites with the linear dimensions ~30–40 nm.

However, such boundaries are not observed in the TEM image of SWCNTs with d ~1.1 nm and length ~15 μm (the upper inserts in Figure 2).

**Figure 3:**
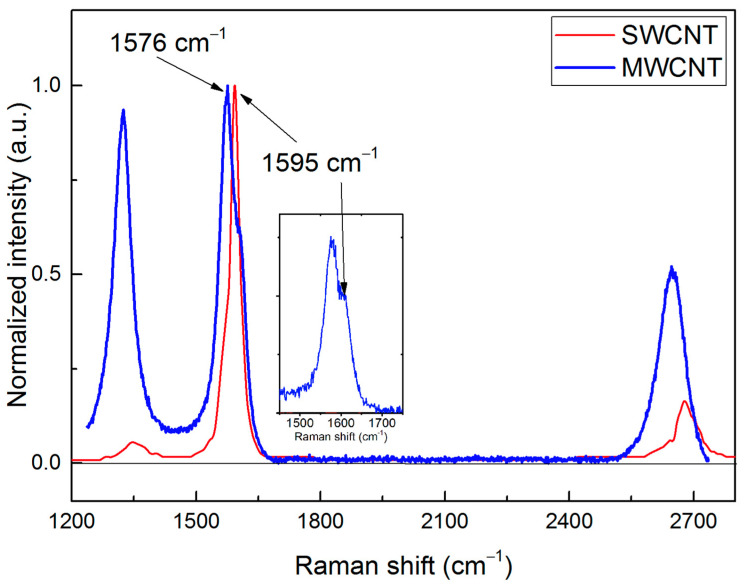
Raman spectra of MWCNTs (blue curve) and SWCNTs (red curve, by Cheap Tubes Inc., Cambridgeport, VT, USA).

Raman spectra for MWCNTs and SWCNTs (by Cheap Tubes Inc., Cambridgeport, VT, USA) in the range from 1200 to 2800 cm^−1^ are shown in Figure 3. The minimum intensity in this range is taken to be zero. The excitation wavelengths are *λ* = 633 nm and λ = 514.5 nm for MWCNTs and SWCNTs, respectively. The main characteristics of Raman spectra are shown in Table 1.

In Raman spectra for SWCNTs (red curve in Figure 3), the narrow G peak near 1595 cm^−1^ corresponding to vibrations of sp^2^-hybridized carbon atoms in the graphene planes is characteristic of an armchair-like structure [34]. Absorption band D (the peak near 1344 cm^−1^, which is associated with defects in the wall structure) and band G′ (the peak near 2675 cm^−1^; an overtone mode of the D band) have low intensity, which indicates a weak defectiveness of the SWCNT structure.

In MWCNTs (blue curve in Figure 3), the wide G peak splits into two peaks at 1576 and 1595 cm^−1^, which indicate the presence of boundaries between the armchair and zigzag graphene fragments (crystallites) [34]. An increase in the D- and G’-peak intensities bears witness to a larger structural disorder in MWCNTs.

The disorder found for MWCNTs is associated with the presence of crystallites of the armchair and zigzag types and boundaries between them. The degree of disorder expressed as the ratio between the *D* and *G* band intensities is related to the size of graphene crystallites $L_{\alpha }$. This value can be calculated by the Tuinstra-Koenig equation [35].

(1) $$L_{\alpha }=\left(2.4\times 10^{-10}\right)\lambda^{4}{\left(\frac{I_{D}}{I_{G}}\right)}^{-1}$$

$L_{\alpha SWCNT}=209\;\mathrm{nm}$ for SWCNTs and for MWCNTs $L_{\alpha MWCNT}=42\;\mathrm{nm}$, respectively. It confirms the armchair-zigzag type of the MWCNT structure.

Structural studies show that MWCNTs prepared by the CVD method have walls with a hexagonal structure formed by armchair and zigzag crystallites. Their linear dimensions are approximately 40 nm. However, there are works [36], where the domain sizes are ~10 nm, which is associated with a stronger structural disorder in the samples.

We consider a possible mechanism of the disordered structure formation in MWCNTs during their synthesis. This mechanism can be associated with the formation of domains of symmetry $D_{2h}$ (armchair) in MWCNTs with the initial symmetry $D_{6h}$ (zigzag) under high temperatures, internal macrodeformation, and mechanical stresses. The formation of armchair-zigzag boundaries was usually considered in terms of energy efficiency [9,10,11].

The formation of such a domain structure is described within the thermodynamic theory of phase transitions [28], which is substantiated by the features of CNT synthesis: cooling, deformation of the hexagonal structure, and residual internal stresses. This means that, apart from possible microscopic displacements and misorientations (microscopic order parameters—$\left\{u_{i}\right\}$) [9], there are macroscopic deformations $\left\{\varepsilon_{ij}\right\}$, interacting with $\left\{u_{i}\right\}$. All these characteristics can lead to the formation of domains in the initial structure. To construct a theory of structural transformation, it is necessary to derive thermodynamic potentials $F_{u}$, $F_{\varepsilon }$, $F_{\mathrm{int}}$, the equations $\frac{\partial F}{\partial \varepsilon }=0$_,_ $\frac{\partial F}{\partial u}=0$ and $u_{i}$, $\varepsilon_{ij}$ for different types of domains using group theory [28].

Assume that $\left\{u_{i}\right\}$ has three components (i=x,y,z), which correspond to a three-ray star of the wave vector with k≠0. Representation D of the wave vector group, which transforms the parameters $\left\{u_{i}\right\}$ will be three-dimensional and will be induced by representation $A_{2u}$ of group $D_{6h}$ [30].

The potentials $F_{u}$ and $F_{\varepsilon }$ for macroscopic deformations of a hexagonal structure can be derived based on [28]

(2) $$F_{u}=\alpha \left(u_{x}^{2}+u_{y}^{2}+u_{z}^{2}\right)+\beta_{1}\left(u_{x}^{4}+u_{y}^{4}+u_{z}^{4}\right)+\beta_{2}\left(u_{x}^{2}u_{y}^{2}+u_{x}^{2}u_{z}^{2}+u_{y}^{2}u_{z}^{2}\right)$$

(3) $$F_{\varepsilon }=\frac{C_{11}}{2}\left(\varepsilon_{xx}^{2}+\varepsilon_{yy}^{2}\right)+\frac{C_{33}}{2}\varepsilon_{zz}^{2}+C_{12}\varepsilon_{xx}\varepsilon_{yy}+C_{13}\left(\varepsilon_{xx}+\varepsilon_{yy}\right)\varepsilon_{zz}$$

where $C_{ij}$—is the elastic moduli and i=x,y,z. Shear strains $\varepsilon_{ij}\;$ in MWCNTs are assumed to be small [37].

To deduce the interaction potential $F_{\mathrm{int}}$ for the parameters $\left\{u_{i}\right\}$ and $\left\{\varepsilon_{ii}\right\}$ we find the eigenfunctions $\left\{u_{i}\right\}$,$\left\{\varepsilon_{ii}\right\}$ which are transformed according to the same representations entering symmetrized squares $\left[D^{2}\right]$ and vector representation $\left[D_{1}^{2}\right]$. These representations are $A_{1g}$ and $E_{2g}$ [30] with the eigenfunctions $\left(u_{x}^{2}+u_{y}^{2}+u_{z}^{2}\right)$, $\left(\varepsilon_{xx}+\varepsilon_{yy}\right)$, $\varepsilon_{zz}\left(A_{1g}\right)$ and $\left(u_{x}^{2}+u_{y}^{2}-2u_{z}^{2}\right)$, $\sqrt{3}\left(u_{x}^{2}-u_{y}^{2}\right)$, $\left(\varepsilon_{xx}-\varepsilon_{yy}\right)\left(E_{2g}\right)$.

Taking into account these functions, we have

(4) $$F_{\mathrm{int}}=\left(u_{x}^{2}+u_{y}^{2}+u_{z}^{2}\right)\left(a_{1}\left(\varepsilon_{xx}+\varepsilon_{yy}\right)+a_{2}\varepsilon_{zz}\right)+b\left(u_{x}^{2}+u_{y}^{2}+u_{z}^{2}\right)\left(\varepsilon_{xx}+\varepsilon_{yy}\right)$$

According to the three axes of the second order $C_{2}{}^{\prime \prime }$ and order parameters $u_{k}=u\;\left(k=1,2,3\right)$, the number of different domains is equal to three. The rest of the components $u_{{\;}_{l\neq k}}$ in each domain are equal to zero. By substituting $u_{k}$ into the equilibrium equation for each domain, we obtain

(5) $$\begin{array}{c} \varepsilon_{xx}^{\left(1\right)}=\varepsilon_{yy}^{\left(1\right)}=\varepsilon_{xx}^{\left(2\right)}=\varepsilon_{yy}^{\left(2\right)}=\varepsilon_{xx}^{\left(3\right)}=\varepsilon_{yy}^{\left(3\right)}=\frac{C_{1}}{2}u^{2}\\ \varepsilon_{zz}^{\left(1\right)}=\varepsilon_{zz}^{\left(2\right)}=\frac{\varepsilon_{zz}^{\left(3\right)}}{2}=\frac{C_{2}}{2}u^{2}, \end{array}$$

where $C_{1}$ and $C_{2}$ are the combinations of the moduli $C_{11}$,$C_{12}$,$C_{13}$,$C_{33}$,

$u^{2}=\alpha^{2}{\left(2\beta_{1}+a_{1}C_{1}+a_{2}C_{2}\right)}^{2}$, and (k)=1,2,3 is the domain number.

Substituting (5) into (4) gives

(6) $$\begin{array}{c} F_{\mathrm{int}}^{\left(1\right)}=F_{\mathrm{int}}^{\left(2\right)}=\left(a_{1}C_{1}+a_{2}\frac{C_{2}}{2}\right)u^{4}\\ F_{\mathrm{int}}^{\left(3\right)}=\left(a_{1}C_{1}+a_{2}C_{2}\right)u^{4} \end{array}$$

It follows from Equation (6) that domains 1 and 2 have the same energy. It means that they always form in pairs and at the same angle to each other along the axes $C_{2}{}^{\prime \prime }$. Domain 3 is independently formed in this three-mode structure and has a direction along the third axis $C_{2}{}^{\prime \prime }$.

The single-domain state in MWCNTs can be obtained at uniaxial pressure. Stresses $\sigma_{ii}$ arising during the synthesis of oriented nanotubes lead to an additional potential interaction of the domains with the elastic field. For each domain separately, this potential has the form

(7) $$F_{\sigma }^{\left(k\right)}=\sigma_{11}\varepsilon_{xx}^{\left(k\right)}+\sigma_{22}\varepsilon_{yy}^{\left(k\right)}+\sigma_{33}\varepsilon_{zz}^{\left(k\right)}$$

Subject to the conditions $\sigma_{33}<0$, $\sigma_{11}=\sigma_{22}=0$, $F_{\sigma }^{\left(3\right)}+F_{\mathrm{int}}^{\left(3\right)}=\left(a_{1}C_{1}+a_{2}C_{2}\right)u^{4}-\sigma_{33}C_{2}u^{2}$ can become minimum at $\left|\sigma_{33}\right|>\frac{a_{2}}{2}u^{2}$, and domains 3 will be the single-mode state. Both domains 1 and 2 will be the single-mode state under other conditions.

Such structures correspond to the experimentally observed armchair domains in zigzag MWCNTs (Figure 2). Splitting of the G peak in the Raman spectrum also indicates such zigzag-armchair domains (Figure 3) [34].

The microscopic formation mechanism $\left\{u_{i}\right\},\;\left\{\varepsilon_{ii}\right\},\sigma_{ii}$, i.e., the geometric rearrangement, is not studied within the thermodynamic description of phase transitions, though there exist works dealing with changes in symmetry, defect evolution as well as the mechanics of formation of new structures [38,39,40].

We model domains of symmetry $D_{2h}$ embedded into the nanotube with symmetry $D_{6h}$ Figure 2, insert). Figure 2 shows that armchair domains intersect fragments of differently oriented zigzag hexagons. It is seen that defects such as pentagons, heptagons, Stone-Wells defects, dangling bonds arise at the boundaries of these domains. The domain structure and the accompanying defects appear due to structural transformation caused by the free energy gain.

Reverse transformation is also possible, i.e., the formation of $D_{6h}$ domains in the original structure of $D_{2h}$, during the relaxation of initial elastic hexagonal strains.

Thus, synthesis conditions for MWCNTs significantly affect their structure and lead to the accumulation of macrostrains and stresses. As a result, thermodynamically equilibrium domains of various hexagonal structures appear in nanotubes. Various structural defects arise at the boundaries of differently oriented domains, which is confirmed by TEM images [36]. This means that boundaries result from the appearance of equilibrium domains rather than domains resulting from the appearance of boundaries [9,10,11].

Structural changes such as the arisen disorder should change all physical properties, including the heat capacity.

**Figure 4:**
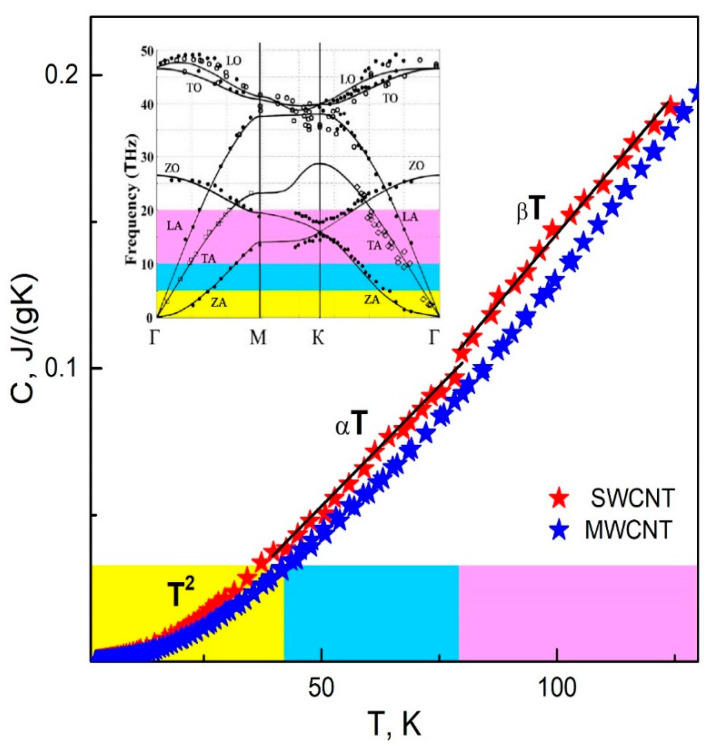
Temperature dependence of the heat capacity of MWCNTs (blue stars) [17] and SWCNTs (red stars) [14]. Phonon spectra of SWCNTs (graphene, solid lines) and MWCNTs (graphite, solid circles, open squares) are given in the insert [41]. A yellow color shows the temperature range from 0 to 40 K, blue from 40 to 80 K, pink from 80 to 150 K, and the corresponding frequency intervals [41].

We estimate the effect of structural changes in MWCNTs on the heat capacity. To do this, we compare the temperature dependences *C*(*T*) for SWCNTs [14] and MWCNTs [17]. This approach agrees also with the data [15,18,19,20] (Figure 4).

The dependences $C\left(T\right)\sim T^{2}$ and $C\left(T\right)\sim T^{3}$ on different temperature intervals are shown in Figure 4. At T>5K, they are similar for both types of nanotubes. At T<5K,$C\left(T\right)\sim T^{3}$ in MWCNTs, whose values are however 10% less than in SWCNTs.

In SWCNTs without the structural disorder, the temperature behavior of the specific heat is determined by the temperature-induced change in the phonon dispersion law. In MWCNTs, the structural disorder is the main feature that determines their properties. Crystallites and their boundaries in MWCNTs scatter phonons, creating dimensional and relaxation effects that influence the formation of physical properties.

The similarity of the dependences *C*(*T*) for SWCNTs and MWCNTs indicates that the formation of boundaries does not cause qualitative changes in the vibration spectrum but produces the size effect, thus limiting phonon wavelengths and leading to local damping.

The analysis of the phonon spectra for SWCNTs and MWCNTs (Figure 4, insert) [41,42] shows that they coincide in the range from 0 to 20 THz (from 0 K to 150 K). This indicates that the difference in *C*(*T*) is only due to the size effect.

Now we enter into a discussion of *C*(*T*) for SWCNTs. We write the internal energy of phonons in the nanotube $E=\int_{-\infty }^{\infty }\frac{\omega \rho \left(\omega \right)}{e^{\omega /T}-1}d\omega$, where ρ(ω)=2ωIm∫0∞kdkω2−ω2(k)+i0 is the phonon density of states [29,43].

According to the analysis of the phonon spectrum [41,42] at ω<5T Hz, ω(k)≈ck, where c is the average speed of sound. At ω>5T Hz, $\omega \left(k\right)\approx \alpha k^{2}$. By substituting the ω(k) values into E, we easily derive the heat capacity $C\left(T\right)=\frac{dE}{dT}$. At, ω<5T Hz $C\left(T\right)\sim T^{2}$, and at ω>5T Hz C(T)~T.

The maximum phonon wavelength for MWCNTs is $\lambda_{\max}\approx L_{\alpha }$, where $L_{\alpha }$ is the crystallite size (unlike $\lambda_{\max}\to \infty$ for SWCNTs). We analyze *C*(*T*) for this case. At low temperatures T→0, $\lambda =L_{\alpha }\frac{T^{*}}{T}\to \infty$, where $T^{*}$ is the temperature corresponding to the change of the dispersion law. This means that phonons are not scattered and $C\left(T\right)\sim T^{3}$, which is experimentally observed (Figure 4).

With increasing temperature $\lambda \to L_{\alpha }$, phonons begin to scatter with the frequency $\omega_{\min}=\frac{1}{\tau }=\frac{c}{L_{\alpha }}$.

By way of example, we calculate the dependence *C*(*T*) in the temperature range 40–80 K, where the flexural vibration modes $\omega \left(k\right)=\alpha k^{2}$ [41,42] and the coefficient $\alpha =H{\left(\frac{E}{12\rho }\right)}^{1/2}$ [44]. The group speed is $\frac{d\omega }{dk}=H{\left(\frac{E}{3\rho }\right)}^{1/2}k$, where H is the layer thickness, E is Young’s modulus, and ρ is the nanotube density. Frequencies of flexural waves forming the heat capacity are in the range from 5 to 10 THz, which, according to the calculations of the phonon spectrum, corresponds to the wave vectors $k=\frac{2\pi }{a}\left(\frac{1}{3}-x,\frac{1}{3\sqrt{3}}-x\right)$ ($x<\frac{1}{4}$). On this interval of wave vectors, we obtain ${\left(\frac{d\omega }{dk}\right)}_{K}\leq 10^{4}\frac{m}{s}$, which agrees with the data from [45]. In MWCNTs, $\frac{d\omega }{dk}$ takes the same values since SWCNTs and MWCNTs have similar phonon spectra.

Calculating the above integral with $\omega \left(k\right)=\alpha k^{2}$ for SWCNTs, we derive

(8) $$C\left(T\right)=\frac{\pi k_{B}{}^{2}S}{3\hbar H}{\left(\frac{3\rho }{E}\right)}^{1/2}T$$

where *S* is the specific surface area, $k_{B}$ is Boltzmann’s constant, and ℏ is Planck’s constant.

For SWCNTs, *E = 1* TPa $\rho =2\;g/cm^{3}$, $S=500–800\;\frac{m^{2}}{g}\;$ [46], H=0.342 nm and $C\approx 8\cdot 10^{-4}T\;\left[J/gK\right]$, which agrees with the experimental data (Figure 4).

For MWCNTs in the same temperature range, the heat capacity is determined by the value $\frac{k_{B}{}^{2}TS}{\pi \hbar \alpha }\left(\xi \left(2\right)-\int_{0}^{\frac{\hbar \omega_{\min}}{kT}}\frac{xdx}{\left(e^{x}-1\right)}\right)$.

At $\omega_{\min}=\frac{c}{\lambda_{\max}}\approx 2.5*10^{11}\;Hz$ and T≈10 K, $\frac{\hbar \omega_{\min}}{k_{B}T}=0.2$.

The second term in the brackets is approximately 10% of ξ(2). This means that the scattering of phonons in crystallites leads to the fact that the heat capacity of MWCNTs is approximately 10% less than that of SWCNTs. In this case, this difference decreases with increasing temperature, which agrees with the experimental data.

## 4. Conclusions

A mechanism for the formation of zigzag-armchair structures in MWCNTs has been proposed in the present paper. The domain structure is formed as a thermodynamic phase transition during the synthesis, with decreasing temperature, hexagonal strains, and residual internal stresses in nanotubes. Linear dimensions of the domains are several tens of nanometers. Such a decrease in the linear dimensions increases the cutoff frequency of phonon scattering and thereby decreases the contribution to the heat capacity of the lattice. Scattering of phonons at the boundaries occurs in the ballistic regime, when the eigenfrequency is more than the scattering frequency ($\omega >\frac{1}{\tau }$). Therefore, the size effect prevails over the relaxation one. A more dramatic situation (hydrodynamic scattering regime) holds in the case of concentration disorder. The revealed dependence of the heat capacity of MWCNTs on the structural disorder allows control over thermal properties of nanotubes and can be useful for the development of thermoelectric, thermal interface materials and nanofluids based on them.

## Figures and Tables

**Table 1:** Characteristics of Raman Spectra of SWCNTs (by Cheap Tubes Inc.) and MWCNTs.

Sample	D (cm^−1^)	G (cm^−1^)	I_D_/I_G_
SWCNTs	1344	1595	0.08
MWCNTs	1325	1576 (1595)	0.9

## Data Availability

The data presented in this study are available on request from the corresponding author.
